# Factors of influence upon overall survival in the treatment of intracranial MPNSTs. Review of the literature and report of a case

**DOI:** 10.1186/1748-717X-5-114

**Published:** 2010-11-24

**Authors:** Konstantinos Gousias, Jan Boström, Attila Kovacs, Pitt Niehusmann, Ingo Wagner, Rudolf Kristof

**Affiliations:** 1 Department of Neurosurgery, University Hospital of Bonn, Sigmund-Freud-Str. 25, Bonn, 53105, Germany; 2 Department of Radiosurgery and Stereotactic Radiotherapy, Mediclin Robert Jancer Clinic, Villenstrasse 4-8, 53129 Bonn, Germany; 3 Department of Neuroradiology, University Hospital of Bonn, Sigmund-Freud-Str. 25, Bonn, 53105, Germany; 4 Department of Neuropathology, University Hospital of Bonn, Sigmund-Freud-Str. 25, Bonn, 53105, Germany; 5 Department of ENT, University Hospital of Bonn, Sigmund-Freud-Str. 25, Bonn, 53105, Germany

## Abstract

**Background:**

Intracranial malignant peripheral nerve sheath tumors are rare entities that carry a poor prognosis. To date, there are no established therapeutic strategies for these tumors.

**Methods:**

We review the present treatment modalities and present the current therapeutic dilemmas. We perform a statistical analysis to evaluate the prognostic factors for Overall Survival of these patients. Additionally, we present our experience with a 64-year-old man with a MPNST of the left cerebellopontine angle.

**Results:**

To our best knowledge, forty three patients with intracranial MPNSTs, including our case, have been published in the international literature. Our analysis showed gross total resection, radiotherapy and female gender to be beneficial prognostic factors of survival in the univariate analysis. Gross total resection was recognized as the only independent predictor of prolonged Overall Survival. In our case, we performed a gross total resection followed for the first time by stereotactically guided radiotherapy.

**Conclusion:**

Considering the results of the statistical analysis and the known advantages of the stereotaxy, we suggest aggressive surgery followed by stereotactically guided radiotherapy as therapy of choice.

## Background

Malignant Peripheral Nerve Sheath Tumors (MPNST) usually arise de novo or from a malignant transformation of a neurofibroma. Rarely MPNSTs may arise from schwannoma, ganglioneuroma or phaeochromocytoma [1,2]. Incidence rates of MPNSTs are identified at less than 1/10^6^/year, with the majority of cases located in the brachial or lumbal plexus. Their intracranial occurrence is even more sporadic. To date, no generally accepted therapeutic strategies or prognostic factors of intracranial MPNSTs are established.

To our best knowledge, 42 cases of intracranial MPNSTs have been reported in the literature, 16 of them concerning the VIIIth nerve [3-13]. We review the applied therapies and identify prognostic factors of OS for these tumors.

Furthermore, we present a case of a MPNST of the VIIIth nerve, and propose a novel therapeutic strategy consisting of aggressive surgical resection followed by stereotactically guided radiotherapy.

## Methods

Twenty case reports and four retrospective clinical studies concerning intracranial MPNSTs were identified using the NCBI PubMed. No limitations regarding the language or time of publication were imposed on the search process. Two studies concerned MPNSTs as a whole, including tumors of the head and neck, without specifying whether the latter were extracranial or intracranial [14,15]. Thus, they were excluded from our review analysis. Similarly excluded were the cases of MPNSTs arising from extracranial trigeminal branches.

Overall survival (OS) was analyzed with the Kaplan-Meier method. Assessments of potential prognostic factors were carried out using log-rank tests. The multivariate analysis was performed using the Cox Regression Hazard Models- Backward Stepwise Procedure. P values ≤ 0.05 were regarded significant.

## Results

A total of forty three patients with intracranial MPNSTs, including our case, were identified. The mean age was 37.6 ± 20.3 (3-69) years. A male predominance (30 males, 69.8%) was observed. 63.9% of the MPNSTs arised de novo; the rest derived from benign tumors. NF1 was present in 17.1% of the patients. Gross total resection (GTR) was achieved in 42.9% whereas 51.3% and 2.3% of the patients received postoperative adjuvant radiotherapy (RT) and chemotherapy, respectively (Table 1-[3-5,7-10,12,13,16-26]). When administrated, radiotherapy was usually whole brain radiation with 60 Gy fractioned over 6 weeks.

**Table 1:** Review of published cases of intracranial MPNSTs

No	Age	Gender	Author, Year [Ref.]	Site	HRT*	NF1	MT*	Resection	RT	Chemo	OS	Death	DM/R*
1	13	M	Ducatman, 1984 [17]	L CN* VII	NR*	no	NR	NR	NR	no	NR	NR	NR
2	18	M	Bruner, 1984 [30]	frontal	NR	no	no	GTR*	no	no	66	no	R
3	15	M	Stefanko, 1986 [21]	L parietooccipital	NR	NR	no	GTR	yes	yes	9	yes	NR
4	24	F	Best, 1987 [31]	R CPA*,	NR	no	no	IR*	no	no	4	yes	NR
5	54	M	Matsumoto,1990 [13]	R CPA, CN VIII	no	no	NR	IR	no	no	4	yes	R
6	47	F	Han, 1992 [32]	R CPA	no	no	no	IR	no	no	11	yes	NR
7	38	M	Maeda, 1993 [33]	R CPA, CN VIII	no	no	no	IR	no	no	2	yes	NR
8	61	F	Singh, 1993 [34]	R cerebellum	NR	NR	no	GTR	yes	no	18	yes	NR
9	8	F	Sharma, 1998 [9]	R temporal lobe	no	no	no	GTR	yes	no	17	no	NR
10	44	M	Comey, 1998 [35]	R CPA, CN VII,VIII	yes	yes	yes	IR	no	no	12	yes	R
11	69	M	Saito,2000 [12]	L CPA, CN VIII	no	NR	NR	IR	no	no	3	no	NR
12	4	F	Tanaka, 2000 [36]	R parietooccipital	NR	no	no	GTR	no	no	19	no	NR
13	30	F	Akimoto, 2000 [37]	L CN V1	no	no	no	IR	yes	no	16	yes	R
14	57	F	Hanabusa,2001 [10]	R CPA, CN VIII	yes	no	yes	IR	yes	no	13	yes	R
15	13	F	Stark, 2001 [38]	L CN V2	no	no	no	GTR	yes	no	14	yes	R
16	36	M	Ueda, 2004 [39]	R+L CN V	no	yes	no	IR	yes	no	10	yes	R
17	43	F	Gonzalez,2007 [11]	L CPA, CN VIII	NR	no	yes	GTR	yes	no	8	yes	M
18	NR	M	Krayenbühl, 2007 [4]	inta- suprasellar	yes	no	yes	IR	yes	no	3	no	no
19	62	M	Miliaras, 2008 [5]	L temporal lobe	no	no	no	GTR	yes	no	13	yes	R
20	40	F	Chibbaro, 2008 [3]	L CN V2	no	no	no	IR	yes	no	21	no	R
21	8	M	Chen, 2008 [7]	L CN V	no	no	yes	GTR	no	no	8	yes	R
22	43	M	Chen, 2008 [7]	L occipital	no	yes	yes	IR	yes	no	4	yes	R
23	3	M	Chen, 2008 [7]	L CN V, CS*	NR	no	no	IR	no	no	4	yes	R
24	35	M	Chen, 2008 [7]	L CN V, CS	NR	no	no	IR	no	no	2	yes	NR
25	46	F	Chen, 2008 [7]	L CN V, CS	NR	no	no	GTR	yes	no	60	no	no
26	62	F	Chen, 2008 [7]	L CPA, CN VII,VIII	NR	no	no	GTR	no	no	4	yes	NR
27	5	M	Chen, 2008 [7]	R V1,orbita	NR	no	no	GTR	no	no	9	yes	NR
28	32	M	Scheithauer, 2009 [8]	R CPA, CN VIII,IX,X,XI	yes	yes	no	IR	yes	no	5	yes	M
29	67	M	Scheithauer, 2009 [8]	R CPA, CN VIII	no	no	yes	IR	no	no	1	yes	NR
30	56	M	Scheithauer, 2009 [8]	R CPA, CN VIII	no	no	yes	IR	no	no	2	yes	R
31	32	M	Scheithauer, 2009 [8]	L CPA, CN VIII	no	yes	no	IR	no	no	3	yes	R
32	26	F	Scheithauer, 2009 [8]	L CPA, CN VII,VIII	no	no	yes	IR	yes	no	NR	NR	NR
33	5	M	Scheithauer, 2009 [8]	L CPA, CN VIII	no	no	no	NR	no	no	NR	NR	NR
34	69	M	Scheithauer, 2009 [8]	R frontal lobe	no	no	no	NR	no	no	4	yes	R
35	50	M	Scheithauer, 2009 [8]	L CN VII	no	NR	yes	GTR	yes	no	17	yes	NR
36	26	M	Scheithauer, 2009 [8]	posterior fossa	NR	NR	NR	NR	NR	no	NR	NR	NR
37	50	M	Scheithauer, 2009 [8]	L CPA	NR	NR	NR	NR	NR	no	36	yes	R
38	30	M	Scheithauer, 2009 [8]	optic chiasma	yes	NR	yes	NR	no	no	2	yes	NR
39	59	M	Scheithauer, 2009 [8]	L gasserion ganglion	NR	NR	NR	NR	NR	no	NR	NR	NR
40	41	M	Scheithauer, 2009 [8]	posterior fossa	NR	no	NR	NR	yes	no	5	yes	R
41	32	M	Scheithauer, 2009 [8]	CN X	yes	yes	yes	IR	yes	no	NR	NR	M
42	62	M	Ziadi, 2010 [40]	L CN V3	no	no	no	GTR	yes	no	17	no	no
43	64	M	present study	L CPA, CN VIII	no	no	yes	GTR	yes	no	12	no	no

*HRT: History of radiation exposure, MT: malignant transformation of a former benign entity (mainly neurofibroma or schwannoma), DM/R: distant metastasis/recurrence, NR: not reported, GTR: gross total resection, IR: incomplete resection, CN: cranial nerve, CPA: cerebellopontine angle, CS: cavernous sinus.

Median OS was 9 months. Progression free survival was not documented in the majority of the cases, and could not be evaluated.

**Figure 1:**
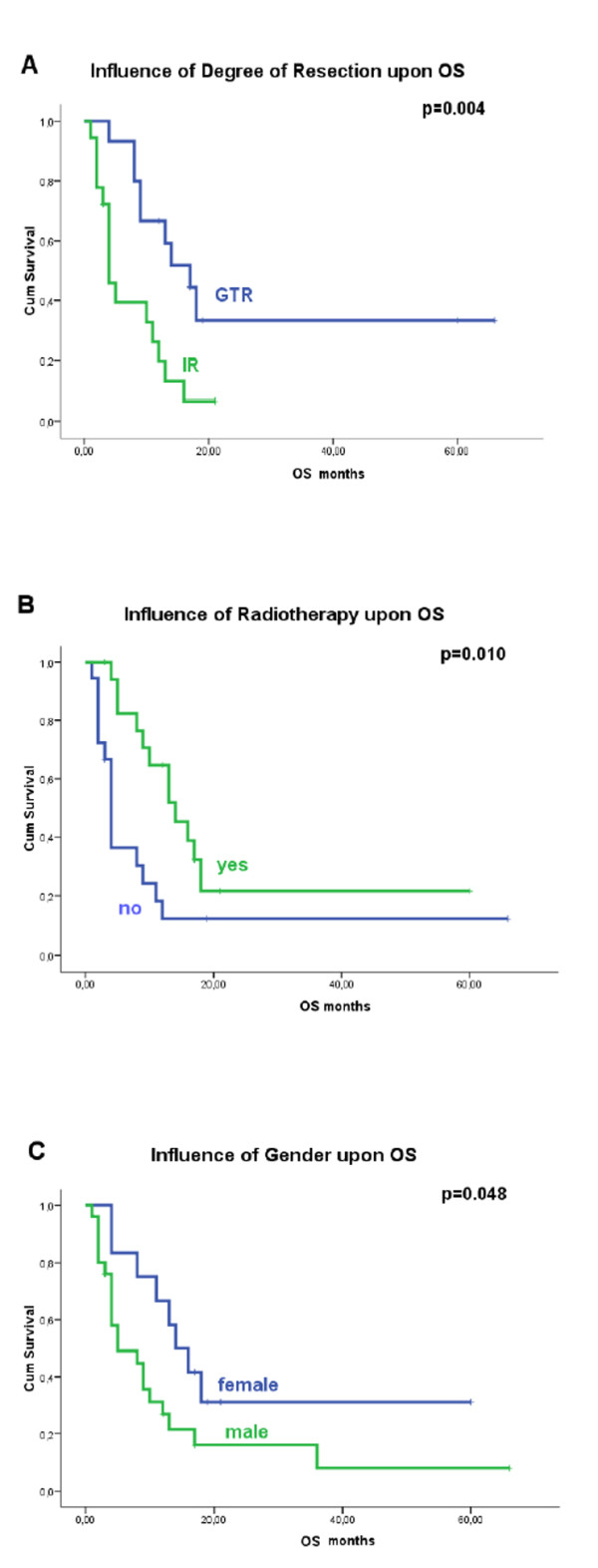
**Kaplan-Meier survival curves showing the influence of, A) degree of resection, B) radiotherapy and C) gender upon Overall Survival**.

In the univariate analysis, female gender (p = 0.048), GTR (p = 0.004) and RT (p = 0.010) were significant beneficial factors for OS (Figure 1). Notably, younger age, malignant transformation of a former benign tumor and the presence of NF1 did not significantly influence outcome (p > 0.05) (Table 2).

**Table 2:** Statistical Analysis

Univariate Analysis*						
	gender	resection	RT	age**	NF1	MT

Log Rank	p	p	p	p	p	p
Overall Survival	**0.048**	**0.004**	**0.010**	0.756	0.132	0.140
Multivariate Analysis ***						
Resection (GTR vs IR)	**p = 0.004**	HR = 0.258	CI 95% (0.102-0.653)			
Gender (female)	p = 0.059	HR = 0.401	CI 95% (0.155-1.037)			

*Kaplan-Meier method and Log Rank test

** over or under 37.6 years old (mean age)

***Cox proportional hazards model

Some factors of potential influence upon OS, such as histological grade and tumour size, were not estimated due to the lack of reported data.

We included the significant factors above in a multivariate analysis, using the backward stepwise procedure. GTR was found to be an independent beneficial prognostic factor for OS (HR = 0.258, CI 95% 0.102-0.653, p = 0.004) (Table 2).

### Illustrative Case

A 64-year-old man presented with progressive headache, vertigo, nausea, hypogeusia and ataxia commencing 3 weeks prior to admission. A left hearing loss was known since three decades. A brain MRI approximately 10 years prior to admission revealed a small tumor localized at the left cerebellopontine angle. There were no history or clinical stigmata of Neurofibromatosis types 1 and 2.

**Figure 2:**
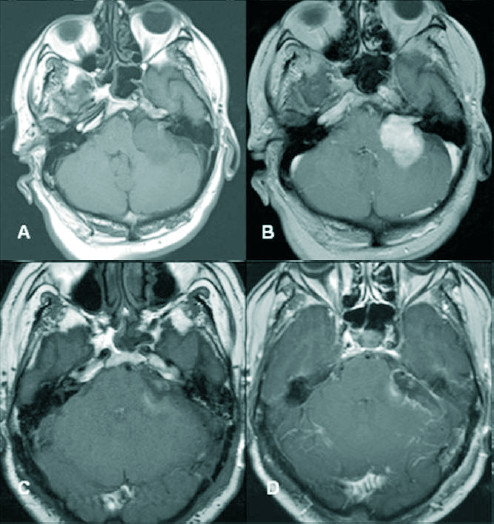
**Preoperative (A+B) and postoperative (C+D) MRIs: (A+C)Axial T1Wse without and (B+D)with contrast**. MRI findings: Enlargement of the left IAC. In non-contrast T1w homogeneous intermediate signal mass in the CPA-IAC cistern on the left with displacement of the middle cerebellar peduncle and strong enhancement after contrast administration. No intramural cysts and no dural tail. C+D, no residual tumor is shown.

Preoperative MRI and CT demonstrate a 3.5*4 cm measuring well delineated contrast-enhancing lesion in the left cerebellopontine angle with mass effect (Figure 2A, B). A thoracoabdominal CT as well as MRI of brachial and lumbal plexus performed ulteriorly excluded other manifestations of the MPNST.

A gross total tumor resection using neuromonitoring of the motor tract and facial nerve function was achieved. Postoperatively, a transient facial nerve palsy House-Brackmann grade III occurred as sole complication.

**Figure 3:**
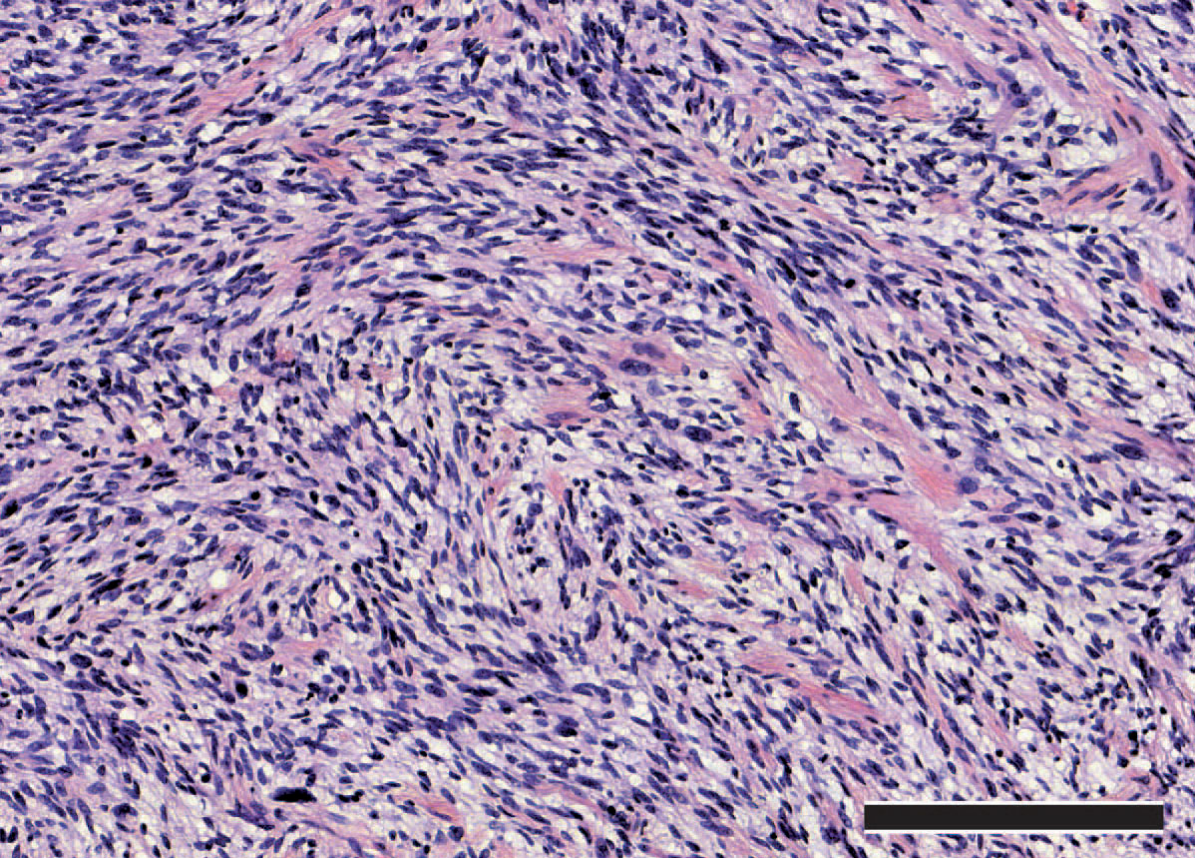
**Histopathological examination revealed a highly cellular tumor with considerable cytologic atypia**. The cytomorphological aspect was dominated by spindle cells with eosinophilic cytoplasm and nuclear enlargement as well as hyperchromasia. Brisk mitotic activity was present, whereas necrosis was no significant feature of the tumor (bar graph - 200 μm).

Histopathological examination revealed a highly cellular tumor with considerable cytologic atypia. (Figure 3). Immunohistochemical examinations revealed only focal immunoreactivity for antibodies against S-100-protein and p75. Tumors cells were strongly immunopositive for vimentin and variable immunoreative for CD99 and Bcl-2. The tumor was classified as grade II according to FNCLCC grading system [27].

**Figure 4:**
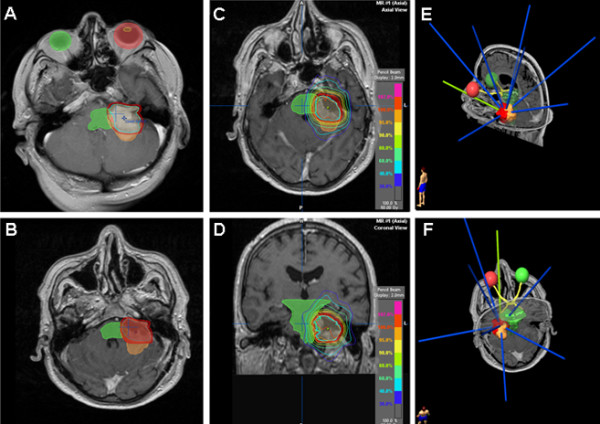
**A)preoperative MRI (tumor brown, CTV blue, PTV red), B)postoperative MRI (tumor brown, CTV blue, PTV red),C)axial and D) coronal MRI showing radiation plan with isodose lines, E and F) non-coplanar and conformal arrangement of the static beams**.

Four weeks after surgery, the patient underwent fractionated stereotactic and image guided radiotherapy using single isocentre dose delivery. A total of 60 Gy was delivered in 30 fractions. The treatment was performed using the Novalis(r) system with micro-multi-leaf-collimator and ExacTrac(r). The patient was immobilized using a relocatable stereotactic frame with an aquaplast mask (all components by BrainLAB(r), Germany). Because there was no detectable residual tumour on post operative MRI (Figure 2C, D), the CTV (clinical target volume) was defined as the former tumour cavity which was delineated by fusing the pre- and post-op T1 MRI sequences with contrast enhancement. The safety margin was set to 2 mm receiving the PTV (planning target volume) of 19.026 cc, (Figure 4A, B). By using 8 non-coplanar conformal static beams the 90% isodose encompassing PTV with a conformity index of 1.52. All delivery parameters were according to the guidelines of RTOG (Figure 4C, D, E, F).

The radiotherapy was well tolerated without acute toxicities. Clinical and MRI follow up at 12 months is without any hints of tumour recurrence.

## Discussion

In contrast to their benign counterparts, neurofibromas or schwannomas, intracranial MPNSTs carry a poor prognosis with a median OS of 9 months, (range 1 to 66 months, present review). In combined series of intracranial and extracranial MPNSTs, Zou et al report a 5-year survival rate of 38.7%, whereas Anghileri et al described a 5-year cause-specific mortality of 39.9%. When the influence of tumor site is considered, Anghileri reported an increased 5-year mortality of head and neck MPNSTs of 66.7%, as compared to 48.8% and 27.5% of trunk and extremities MPNSTs, respectively. The rarity of intracranial MPNSTs hampers the establishment of evidence based strategies for their optimal treatment. Thus, the management of the intracranial MPNSTs should also consider the experience gained from the treatment of extracranial MPNSTs.

Anghileri et al conducted a study of 205 patients with MPNSTs, of which 9 cases were head and neck tumors, and found that GTR, achieved in 62% of the patients, correlated significantly with longer OS, and inversely with local recurrence on multivariate analysis [14]. Zou et al carried out another study of 140 patients with MPNSTs, including 20 tumours of the head and neck, and showed that a complete surgical resection was inversely related to local recurrence on univariate analysis [15]. The results of the present review verify for intracranial MPNSTs the statistically significant influence of GTR upon OS in the univariate and multivariate analysis. Thus, a main goal in the treatment of the intracranial MPNSTs should be the complete surgical tumour resection with preservation of neurological function, whenever applicable.

The role of adjuvant radiotherapy remains controversial. Some studies suggest that radiation may be implicated in the pathogenesis of MPNSTs [8,28].Foley et al suggested that ionizing radiation may cause chromosomal injury and induce proliferation as well as cytologic atypia in Schwann cells, resulting in radiation-induced MPNSTs [29].In our review series, 41.7% of patients harbouring a malignant transformation to MPNST received radiation in their history. Other studies haven't shown any positive effects of radiotherapy on patients outcome[30-32], while the recent literature indicates the beneficial role of the radiotherapy in local control of disease after a total or a near total resection of extracranial MPNSTs [14,33-38]. Anghileri et al found adjuvant radiotherapy to be significantly related to longer OS on multivariate analysis, while no correlations with local recurrence or distant metastases were observed [14]. The radiation dosage administrated in the majority of the cases was 50 - 60 Gy. Our review revealed the beneficial prognostic significance of adjuvant radiotherapy for OS in the univariate analysis. However, the multivariate analysis failed to show an independent influence of RT on OS. This could be related to the limited sample of patients. Considering the above findings and the highly malignant histological appearance of the tumour, in our patient we decided for adjuvant radiotherapy with stereotactic guidance due to its precise dosage delivery while sparing the adjacent healthy brain tissue. This strategy provides the possibility to apply an adequate high dose of 60 Gy despite of nearby sensitive risk structures like the brainstem. Thus, we were able to take advantages of both stereotactic radiotherapy and conventional fractionation while minimising the risks of RT-inducing brain injury like radiation necrosis and cognitive decline.

The optimal radiation dose has not yet been defined. We decided for a total dose of 60 Gy balancing the relatively high radiation dose to the highly malignant histological tumour appearance.

Some authors consider MPNSTs to be chemotherapy-resistant [28] while others suggest that surgery followed by combined radiochemotherapy results in improved survival [39]. Two recent studies of large series of peripheral MPNSTs failed to show any benefit of chemotherapy [7,34]. Therefore, in our patient, chemotherapy was decided to be spared for the case of tumour relapse or metastatic disease.

In the present patient the MPNST seems to have resulted from the malignant transformation of a pre-existing benign schwannoma. 36.1% of the review cases experience a progression of benign tumor to malignancy, having a worse OS compared to MPNSTs arising de novo. The latter difference though did not reach statistic significance (8.46 vs 22.95 months, p = 0.140). These observations point out the importance of a thorough long-time follow-up of all benign intracranial schwannomas and neurofibromas that have not been resected. However, it is not clear whether MRI follow-up can reliably indicate the exceptional transition of a schwannoma to a MPNST. Approximately, 25 to 50% of MPNSTs are associated with NF-1. The overall lifetime risk of genesis of MPNST in patients with NF-1 is estimated to be from 8 to 13% [14,40]. In the present review 17.1% of intracranial MPNSTs were related to NF-1.

It is noteworthy, that the female gender is less likely to present with intracranial MPNST and that females harbouring this tumour have a significant longer OS than men. Further studies are needed to enlighten the background of these observations.

## Conclusion

In conclusion, we propose as therapeutic strategy for intracranial MPNST consisting of the maximal surgical resection feasible with preservation of neurological function, followed by adjuvant stereotactically guided radiotherapy. This strategy minimises the possible complications of surgery as well as of brain radiation. Chemotherapy should probably be spared for relapsed or metastasized disease.

## Abbreviations

CTV: Clinical target volume; GTR: Gross total resection; MPNST: Malignant peripheral nerve sheath tumor; NF1: Neurofibromatosis 1; OS: Overall survival; PTV: Planning target volume; RTOG: Radiation therapy oncology group for stereotactic radiotherapy.

## Competing interests

The authors declare that they have no competing interests.

## Authors' contributions

All of the authors have been involved in drafting this paper and have read and approved the final manuscript. KG conceived the idea of the paper, reported the case, performed the literature research and statistical analysis, wrote the paper, was the attendant physician-resident during the stay of the patient at Hospital and follow up the patient through tel.interviews each month. JB managed the patient concerning the stereotactically guided radiotherapy (in another clinic), wrote the part of the paper concerning radiotherapy and followed up the patient at his out-patient clinic. AK was the radiologist performing the preoperative and postoperative CT and MRI scans and wrote the part of the paper concerning the illustrations. PN was the pathologist who examined the tissue and wrote the part of the pathology evaluation. IW performed the ETN examination preoperatively and postoperatively, as well as performed with KG the relevant literature research. RK was the neurosurgeon who operated the patient, was the supervisor of the clinic admitted the patient, decided for the therapy procedures and revised the manuscript. All authors read and approved the final draft.
